# Diagnostic efficacy of soluble ST2 in pediatric fulminant myocarditis

**DOI:** 10.3389/fped.2025.1417341

**Published:** 2025-03-03

**Authors:** YanZhu Huang, YiHu Lin, MingHong Fu, WeiFeng Zhang

**Affiliations:** ^1^ Department of Pediatric Neurology, Quanzhou Women and Children’s Hospital, Fujian, China; ^2^ Department of Pediatric Intensive Care Unit, Quanzhou Women and Children’s Hospital, Fujian, China; ^3^ Department of Pediatric Cardiovascular, Quanzhou Women and Children’s Hospital, Fujian, China; ^4^ Department of Neonatology, Quanzhou Women and Children’s Hospital, Quanzhou, Fujian, China

**Keywords:** ST2, pediatric, fulminant myocarditis, inflammation, diagnostic test

## Abstract

**Background and aims:**

Early identification of fulminant myocarditis (FM) is the key to reducing mortality, but there is still a lack of effective biomarkers for diagnosis. The aim of this study was to investigate the value of soluble ST2 (sST2) in identifying FM in children.

**Methods:**

This was a single-center clinical observational study. We consecutively enrolled 144 children younger than 14 years of age diagnosed with viral myocarditis between January 2018 and November 2023, of whom 63 were diagnosed with FM.

**Results:**

The sST2 level in the FM group was significantly higher than that in the non-FM group [104.40 (68.80, 150.10) vs. 38.30 (19.85, 55.05), *p* < 0.001]. ROC curves showed that the optimal cut-off values of sST2, TNI, NT-proBNP and CRP for FM were 63.8 ng/ml, 13.3 ng/ml, 3182 pg/ml and 26.5 mg/L, respectively. The sensitivity and specificity of sST2 were 84.13% and 88.9%, indicating the highest early diagnosis efficiency. Multifactorial correction showed that sST2 ≥ 63.8 ng/ml and NT-proBNP ≥ 3182 pg/ml were independent diagnostic predictors of FM (OR = 22.374, 95% CI: 8.140 ∼ 61.499, *P* < 0.001), and (OR = 3.208, 95% CI: 1.163 ∼ 8.846, *P* = 0.024).

**Conclusions:**

With high sensitivity and specificity, sST2 may serve as a strong predictor of pediatric FM.

## 1 Introduction

Acute myocarditis is a kind of myocardial inflammatory disease caused by multiple etiologies, usually caused by viral infection, a few can be caused by autoimmune diseases, toxins and drugs (1). In pediatric patients, its severity ranges from mild symptoms with subclinical myocardial involvement to a fulminant course with cardiogenic shock, life-threatening arrhythmias and even death (2–4). Fulminant myocarditis (FM) is the most serious type of acute myocarditis, characterized by an acute onset and rapid progression (2, 5). Despite recent advances in treatment, the mortality rate of FM in children remains high, ranging from 48.4% in Japan to 9% in France (6, 7). Thus, how to identify and diagnose FM as early as possible is the key link to formulate effective intervention measures and improve prognosis (8).

Currently, the diagnosis of pediatric acute myocarditis partly depends on circulating troponin I (TNI), N-terminal pro-B-type natriuretic peptide (NT-proBNP) and echocardiography (8–10). Endomyocardial biopsy is the gold standard for the diagnosis of myocarditis. However, this method is based only on localised myocardial pathology and is therefore of limited value in diagnosing FM with extensive myocardial tissue damage (8). Although contrast-enhanced cardiac magnetic resonance imaging is a more sensitive technique for detecting the extent of myocardial damage, its use may be limited and not easily accessible in emergency situations (11).

Given the rapid progression and changes in patients with FM and the poorer prognosis compared to non-FM, there is an urgent need to find new simple and reliable biomarkers to distinguish between the two (12, 13). With further understanding of the pathogenesis of myocarditis, FM is considered to be a severe inflammatory disease of the heart and therefore inflammation-related cytokines may serve as potential biomarkers (14). One study comprehensively analyzed 122 inflammatory cytokines in the plasma of adult FM patients at admission and discharge and found that soluble suppression of tumorigenicity 2 (sST2) protein showed the most significant changes during disease onset and regression, confirming sST2 as a highly specific and sensitive biomarker for FM during acute episodes of the disease (15). Unfortunately, myocarditis in children is more susceptible to fulminant onset and there is a lack of validated indicators to predict diagnosis (8). The aim of this study was to analyse the correlation between sST2 and other generic clinical indicators and to evaluate the efficacy of sST2 in the early diagnosis of pediatric FM.

## 2 Material and methods

### 2.1 Study population

Children hospitalised between January 2018 and November 2023 who met the following criteria were enrolled: less than 14 years; consistent with the diagnosis of acute viral myocarditis (16); and exclusion of COVID-19 infection, toxic myocarditis, rheumatic myocarditis, congenital heart disease, primary or secondary cardiomyopathy, congenital atrioventricular block, hyperthyroid cardiomyopathy, metabolic disease or drug-related myocardial damage. The families of the children were informed of this study and signed the informed consent. All methods were carried out in accordance with approved guidelines and regulations. The trial was approved by the Ethics Committee of the Quanzhou Women and Children's Hospital.

### 2.2 Research methods and data collection

The enrolled children with acute viral myocarditis were categorized as fulminant or non fulminant based on clinical features and presentations. Specifically, the diagnostic criteria for FM included: rapid onset of acute heart failure symptoms, life-threatening arrhythmias, severely impaired left ventricular function, or cardiogenic shock in less than 2 weeks in the absence of coronary artery disease or other known etiology; positive inotropic support or mechanical circulatory support due to hemodynamic instability (16). Data collection: ① General information included demographic data, past medical history, prodromal symptoms, clinical signs, routine haematology and electrocardiogram on admission. ② Serum sST2 assay. 2 ml of venous blood was collected within 24 h of admission, placed in sodium heparin tubes, centrifuged at 4,000 rpm/min for 10 min, and the serum was separated and stored in a −80°C refrigerator for testing. Circulating levels of human sST2 were measured using the soluble ST2 (sST2) BioAssay™ ELISA Kit (Human) from Usbiological (catalog number: 530074) according to the manufacturer's instructions. ③ Admission cardiac ultrasound examination. Echocardiography was performed within 24 h of admission.

### 2.3 Statistical analysis

Normality of all continuous variables was tested using the Kolmogorov–Smirnov method. Continuous variables with normal distribution were expressed as mean ± standard deviation (¯x ± s) and independent samples *t*-test was used for comparison between groups. Continuous variables with non-normal distribution were expressed as median and quarterly interval (Q1, Q3) and compared using Mann–Whitney test. Categorical variables were presented in frequency and compared using chi-square tests. The correlation between serum sST2 and other clinical indicators was analyzed by linear regression. Receiver operating characteristic (ROC) curve was used to analyze the best predictive cut-off values of pediatric FM by sST2, TNI, NT-proBNP and c-reactive protein (CRP). Logistic regression analysis was used to determine the independent clinical predictors of FM. The MedCalc statistical software was used to plot the area under the curve (AUC) and to perform Delong tests to compare the differences between the aforementioned indicators. SPSS 26.0 software (IBM, Armonk, NY) was used to test residual statistical analysis. *P* < 0.05 was considered statistically significant.

## 3 Results

### 3.1 Comparison of characteristics between non-FM and FM

A total of 144 cases of acute viral myocarditis were divided into two groups: FM and non-FM. Fatigue in prodrome, amaurosis/syncope in clinical symptoms, TNI, NT-proBNP, CRP, sST2, creatinine (Cr) and the proportion of ventricular tachycardia on admission were all higher in FM group than those in non-FM group (*P* < 0.05), while the proportion of normal electrocardiogram, admission left ventricular ejection fraction (LVEF) and LVEF ≤ 40% proportions were lower than those in non-FM group (*P* < 0.05), and the differences in the remaining clinical indicators were not statistically significant (Table 1).

**Table 1:** Baseline characteristics of pediatric patients.

Item	non-FM (*n* = 81)	FM (*n* = 63)	*χ*2、z or t	*P*-value
Age (yr)	5.75 ± 2.84	6.49 ± 2.66	−1.593	0.113
Male/female (*n*/*n*)	41/40	36/27	0.607	0.436
Prodromal symptoms, *n* (%)				
Fever	65 (80.25)	57 (90.48)	2.865	0.091
Gastrointestinal symptoms	15 (18.52)	9 (14.29)	0.457	0.499
Respiratory symptoms	6 (7.41)	10 (15.87)	2.571	0.109
Fatigue	4 (4.94)	11 (17.46)	4.800	0.028
Other (dizziness, etc.)	3 (3.70)	4 (6.35)	0.117	0.733
Presenting symptoms, *n* (%)				
Chest tightness/chest pain	48 (59.26)	41 (65.08)	0.509	0.476
Dyspnea	15 (18.52)	18 (28.57)	2.207	0.154
Amaurosis/syncope	1 (1.23)	9 (14.29)	7.430	0.006
Vomiting/abdominal pain	7 (8.64)	9 (14.29)	1.143	0.285
Other (crying and irritability, etc.)	11 (13.58)	3 (4.76)	2.215	0.137
Ematological examination				
TNI (ng/ml)	10.00 (7.30,12.90)	13.50 (9.80,16.10)	−4.366	<0.001
NT-proBNP (pg/ml)	2,426.00 (1,916.50,2,991.50)	4,880.00 (2,540.00,7,258.00)	−4.992	<0.001
CRP (mg/L)	20.70 (12.55,35.80)	31.60 (18.80,46.20)	−4.001	<0.001
sST2 (ng/ml)	38.30 (19.85,55.05)	104.40 (68.80,150.10)	−7.950	<0.001
Cr (μmol/L)	63.47 ± 14.41	81.41 ± 19.68	−6.312	<0.001
Admission electrocardiogram, *n* (%)				
ST-T abnormalities	53 (65.43)	42 (66.67)	0.024	0.877
Frequent premature beats	12 (14.81)	5 (7.94)	1.160	0.204
Atrioventricular block	1 (1.23)	5 (7.94)	2.484	0.115
Ventricular tachycardia	0 (0.00)	7 (11.11)	/	0.003
Supraventricular tachycardia	5 (6.17)	2 (3.17)	0.193	0.660
Normal	14 (17.28)	2 (3.17)	5.786	0.016
Admission echocardiogram				
LVEF (%)	52.93 ± 8.37	46.24 ± 8.46	4.735	<0.001
LVEF ≤ 40%, *n* (%)	7 (8.64)	21(33.33)	13.793	<0.001

non-FM, non-fulminant myocarditis; FM, fulminant myocarditis;TNI, troponin I; NT-proBNP, N-terminal pro-B-type natriuretic peptide; CRP, c-reactive protein; sST2, soluble suppression of tumorigenicity 2; Cr, Creatinine; LVEF, left ventricular ejection fraction.

### 3.2 Analysis of the differences in sST2 and correlation with traditional clinical indicators between non-FM and FM

**Figure 1:**
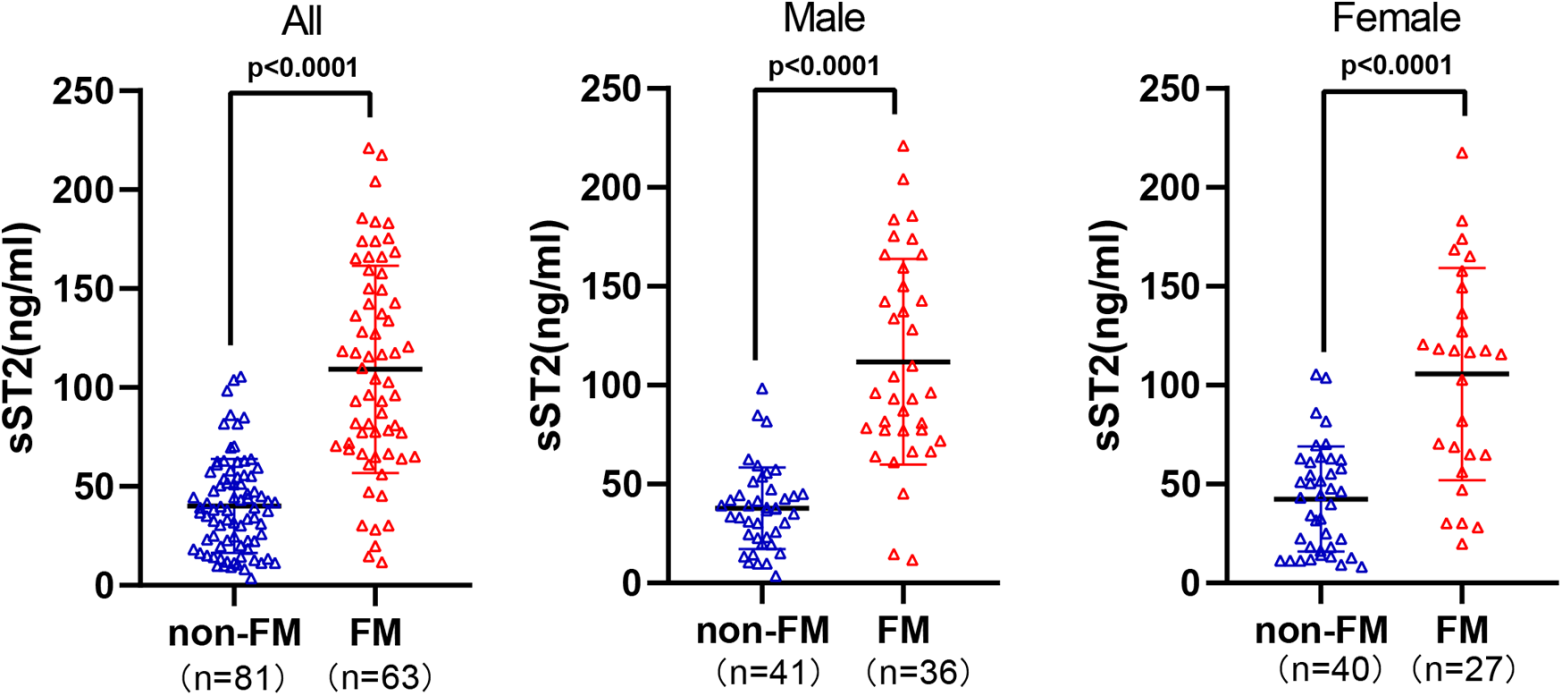
Differences in sST2 between all, male, female children with non-FM and FM.

**Figure 2:**
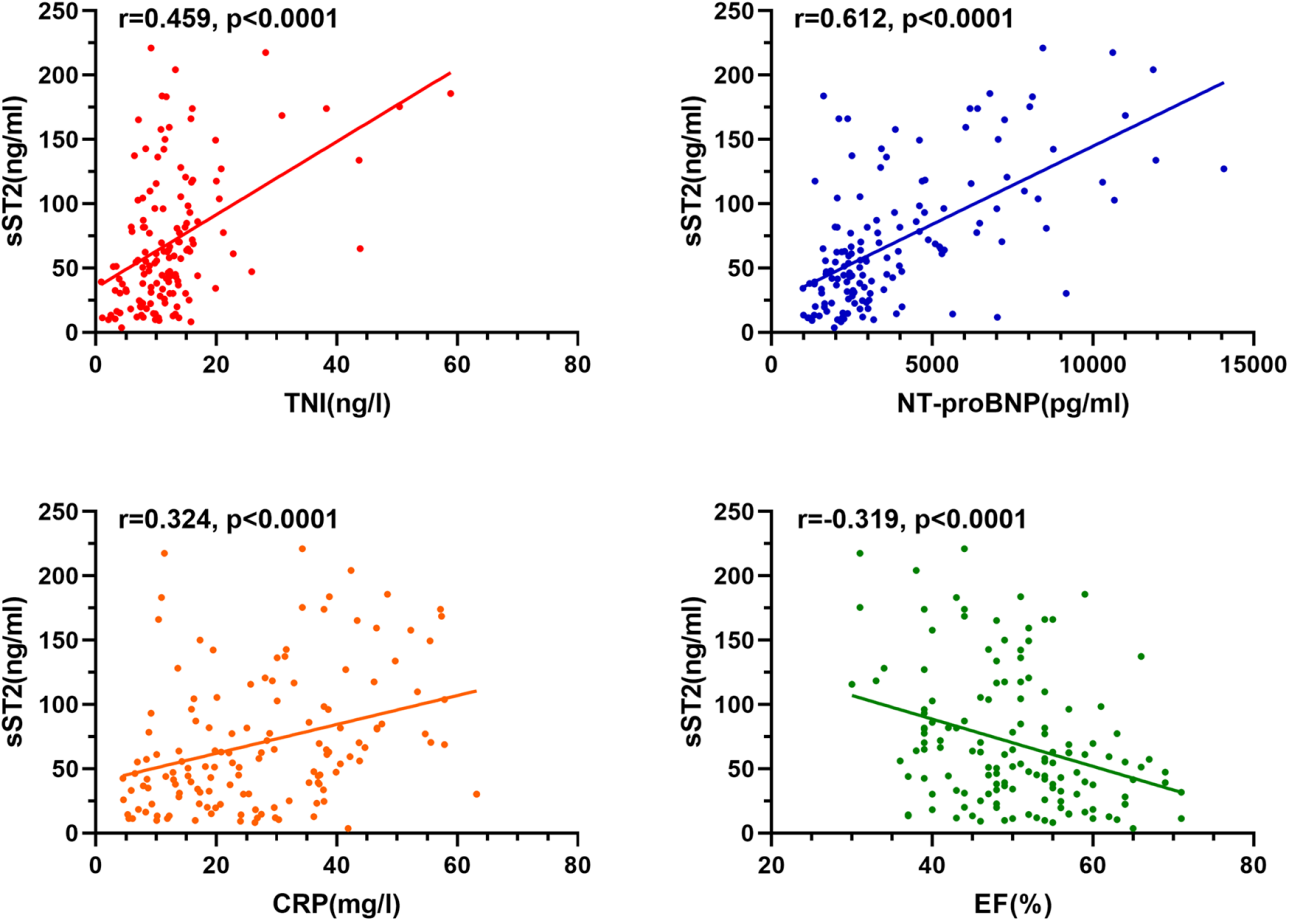
Correlation analysis of sST2 with TNI, NT-proBNP, CRP and LVEF.

In all children, male children and female children, sST2 was significantly higher in FM group than in non-FM group (*P* < 0.05) (Figure 1). Further linear correlation analysis of the data from all enrolled subjects revealed that the positive correlations of sST2 with traditional clinical indicators were ranked in order of NT-proBNP (*r* = 0.612, *P* < 0.001), TNI (*r* = 0.459, *P* < 0.001), CRP (*r* = 0.324, *P* < 0.001), and negatively correlated with LVEF (*r* = −0.319, *P* < 0.001) (Figure 2).

### 3.3 Diagnostic efficacy of plasma sST2 in pediatric FM

**Figure 3:**
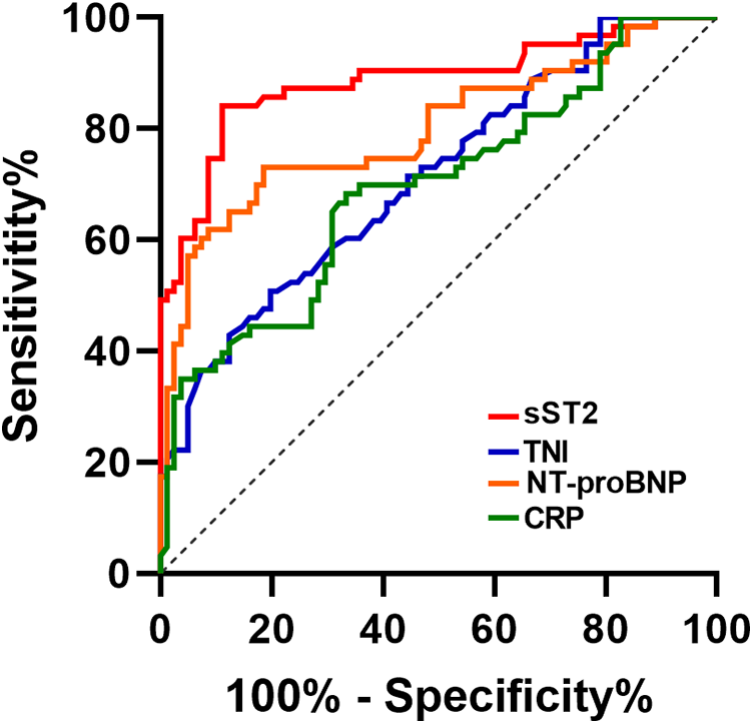
ROC curve of sST2, TNI, NT-proBNP and CRP in diagnosis of FM.

The ROC curves of sST2, TNI, NT-proBNP and CRP for pediatric FM diagnosis were plotted (Figure 3). The results showed that the optimal cut-off value of sST2 was 63.8 ng/ml with an AUC of 0.887, and its diagnostic efficacy was superior to that of TNI, NT-proBNP and CRP (Table 2). The DeLong test was used to compare the AUC difference between sST2 and the other three indicators, and it was found that the AUC of sST2 was higher than that of TNI (difference between areas = 0.174, *P* < 0.001), NT-proBNP (difference between areas = 0.088, *P* = 0.042), and CRP (difference between areas = 0.192, *P* < 0.001).

**Table 2:** Diagnostic value of sST2, TNI, NT-proBNP and CRP in pediatric FM.

Diagnostic indicator	AUC	Cut-off	Sensitivity (%)	Specifcity (%)
sST2	0.887	63.8	84.13	88.89
TNI	0.712	13.3	50.79	80.25
NT-proBNP	0.799	3,182	73.02	81.48
CRP	0.695	26.5	68.25	66.67

sST2, soluble suppression of tumorigenicity 2; TNI, troponin I; NT-proBNP, N-terminal pro-B-type natriuretic peptide; CRP, c-reactive protein; FM, fulminant myocarditis; AUC, area under curve.

### 3.4 Multifactorial logistic regression analysis of pediatric FM

Age, gender, renal insufficiency (Cr ≥ 69 μmol/L), sST2 ≥ 63.8 ng/ml, TNI ≥ 13.3 ng/L, NT-proBNP ≥ 3182 pg/ml, CRP ≥ 26.5 mg/L and LVEF ≤ 40% were introduced into the logistic regression model and analyzed by applying the forward stepwise method. Multivariate adjustment revealed that both sST2 ≥ 63.8 ng/ml and NT-proBNP ≥ 3182 pg/ml were independent diagnostic predictors of FM in hospitalized children (OR = 22.374, 95% CI: 8.140–61.499, *P* < 0.001), (OR = 3.208, 95% CI: 1.163–8.846, *P* = 0.024) (Table 3).

**Table 3:** Logistic regression analysis of acute myocarditis during hospitalization.

Items	Univariate analysis		Multivariate analysis	
	Odds ratio (95% CI) *P*-value		Odds ratio (95% CI) *P-*value	
Age	1.115 (0.915–1.359)	0.279		
Gender	1.746 (0.588–5.181)	0.315		
Renal insufficiency (Cr ≥ 69 μmol/L)	3.367 (1.100–10.305)	0.033		
sST2 ≥ 63.8 ng/ml	16.593 (5.246–52.479)	<0.001	22.374 (8.140–61.499)	<0.001
TNI ≥ 13.3 ng/L	1.404 (0.429–4.593)	0.574		
NT-proBNP ≥ 3182 pg/ml	1.709 (0.529–5.520)	0.037	3.208 (1.163–8.846)	0.024
CRP ≥ 26.5 mg/L	2.462 (0.860–7.049)	0.093		
LVEF ≤ 40%	2.713 (1.328–5.545)	0.048		

Cr, Creatinine; sST2, soluble suppression of tumorigenicity 2; TNI, troponin I; NT-proBNP, N-terminal pro-B-type natriuretic peptide; CRP, c-reactive protein; LVEF, left ventricular ejection fraction.

## 4 Discussion

To our knowledge, this is the first study to assess the diagnostic value of sST2 in pediatric FM. In this study, we found that sST2 was significantly higher in FM than in non-FM regardless of gender and had better diagnostic efficacy than TNI, NT-proBNP, and CRP, making it a highly sensitive and specific predictor for the early assessment of FM in children.

FM is a severe form of viral myocarditis, a disease of rapid onset and progression with a wide range of clinical manifestations and no specific laboratory indications, often associated with severe haemodynamic disturbances requiring early mechanical circulatory support to maintain tissue perfusion (17). During the onset phase of FM, many types of risk-related molecular patterns are produced and released into the bloodstream that can mediate inflammatory responses and organ failure (18, 19). In the most comprehensive clinicopathological description of myocarditis, researchers from Johns Hopkins University were the first to report that the fulminant presentation of patients with myocarditis may be a marker of a more robust inflammatory/immune response that could predict more effective viral clearance, supporting the presence of so-called inflammatory storms as part of the disease's pathogenic features (20). Therefore, the search for possible FM diagnostic biomarkers from the perspective of inflammatory factors has gradually received the attention of the academic community (21, 22). ST2 is a member of the interleukin 1 receptor family and comprises two isomers, soluble ST2 (sST2) and the transmembrane form of ST2 (ST2l) (23). From a cardiovascular perspective, sST2 protein is over expressed in fibroblasts and cardiomyocytes under different conditions and various triggers (23, 24). As a comprehensive indicator reflecting the degree of mechanical stress stimulation and inflammation, sST2 has become one of the new biomarkers for a variety of cardiovascular diseases (24, 25).

In this study, an analysis of 144 pediatric patients with acute viral myocarditis revealed that children with FM had more prodromal symptoms of fatigue, clinical symptoms of syncope or amaurosis and ventricular tachycardia compared with non-FM children. Meanwhile, TNI, NT-proBNP, CRP, and Cr were higher in the FM group than in the non-FM group, whereas the proportions of normal electrocardiograms, admission LVEF and LVEF ≤ 40% were lower than those in the non-FM group. The above general data suggest that low levels of routine haematological indicators, a low reduction in ejection fraction and a normal admission ECG may be initial clues to a non-fulminant episode, while the presence of syncope or amaurosis and evidence of ventricular tachycardia in the clinical presentation may suggest the possibility of a fulminant episode, but all of the above indicators lack diagnostic accuracy (10, 26–28).

To this end, this study focused on sST2, and the data statistics showed that sST2 was higher in the FM group than in the non-FM group regardless of gender. It has been demonstrated that elevated plasma sST2 is not only a reflection of the systemic inflammatory response, but also a result of local cardiac stress and increased mechanical stress; therefore, FM patients with significant inflammatory storm and extensive myocardial injury with increased mechanical stress may mediate higher sST2 levels (5, 15). Interestingly, linear correlation analysis revealed that sST2 was positively correlated with the inflammatory storm factor CRP, the mechanical stress indicator NT-ProBNP, and the biomarker of myocardial injury TNI, and negatively correlated with LVEF, which seems to provide further support for the apparent increase in sST2 in FM based on the perspective of pathophysiologic mechanisms of the inflammatory response, increased mechanical stress associated with cardiac injury, and also suggests that sST2 may reflect the disease condition from multiple dimensions. Further ROC curve analysis of sST2, TNI, NT-proBNP and CRP showed that when the optimal threshold value of sST2 was set at 63.8 ng/ml for the diagnosis of FM, the AUC was 0.887, the sensitivity was 84.13% and the specificity was 88.89%, all of which were better than the other three indicators. A recent study, which recruited 4 healthy controls and 4 sex- and age-matched patients with FM, found that nearly one-third of circulating inflammation-associated cytokines [39 of 122 (32.0%)] were significantly altered in blood samples of patients with FM upon admission; of these, sST2 showed the most significant changes during the onset and regression of FM. In the cohort study, a plasma sST2 threshold of 58.39 ng/ml was found to diagnose FM with 92.3% accuracy, confirming the ability of this indicator to distinguish FM with higher specificity and sensitivity than currently established biomarkers (15). Our study had similar data thresholds with an optimal cut-off value of 63.8 ng/ml, further confirming the diagnostic efficacy of sST2 for pediatric FM and solidifying the evidence in the pediatric field.

In order to determine whether multiple clinical parameters have independent predictive value in the diagnosis of pediatric FM, multivariate binary logstic regression analysis was performed, with only sST2 and NT-proBNP retained in the final equation. Most pediatric FM develops rapidly as a clinical manifestation of acute heart failure and is the result of extensive inflammatory damage to the myocardium (8). NT-proBNP increases in the presence of myocardial dysfunction, and the fact that common causes of myocardial injury and dysfunction, such as coronary artery disease, are very rare in children compared to adults, which makes NT-proBNP even more valuable (29). In a single-centre retrospective study investigating children with FM, data showed that higher peak BNP levels were an independent risk factor for cardiac arrest or mechanical circulatory support, indicating the value of NT-proBNP in paediatric FM (30). The present study further identified NT-proBNP as an independent predictor for differentiating pediatric FM from non-FM as well. As mentioned previously, mechanical stress and inflammation are the two main causes of elevated sST2 levels (24). Thus, sST2, in addition to reflecting mechanical stress like BNP, can more fully respond to systemic inflammatory storm transitions and may be part of the reason why sST2 is superior to NT-proBNP in terms of diagnostic efficacy. Moreover, age, body mass index and renal function have less influence on it, further supporting the prospect of the predictive diagnostic application of sST2 in cardiovascular diseases, including pediatric FM in this study (31, 32). However, there are some limitations to this study. First, this study is a single-centre, observational study with a limited sample size, which affects the generalisability of the results. Second, the absence of cardiac magnetic resonance imaging resulted in a lack of precision in the diagnosis of some myocarditis. Finally, due to data limitations, the relevance of sST2 in the development of dilated cardiomyopathy after myocarditis and its impact on long-term prognosis could not be analysed, which needs further study.

## 5 Conclusion

In acute myocarditis, sST2 was significantly better than those of TNI, NT-proBNP and CRP in predicting FM. sST2, as a strong independent predictor for the diagnosis of pediatric FM, has a high diagnostic efficacy and deserves further clinical exploration and promotion.

## Data Availability

The original contributions presented in the study are included in the article/Supplementary Material, further inquiries can be directed to the corresponding author.
